# A Case of Congenital Hepatic Fibrosis Associated With Medullary Sponge Kidney-Radiologic and Pathologic Features

**DOI:** 10.4021/gr397w

**Published:** 2012-03-20

**Authors:** Lei Zhu, Gang Zhao, Chong-Fu Jia, Yan Li

**Affiliations:** a Department of Gastroenterology, The First Affiliated Hospital, Dalian Medical University, Dalian City, 116011, Liaoning Province, China; b Chong-Fu Jia, Cardiovascular Radiology Center, The First Affiliated Hospital, Dalian Medical University, Dalian City, 116011, Liaoning Province, China; c Department of Gastroenterology, Shengjing Hospital, China Medical University, Shenyang City, 110001, Liaoning Province, China

**Keywords:** Congenital hepatic fibrosis, Medullary sponge kidney, Radiology, Pathology

## Abstract

Congenital hepatic fibrosis is an exceedingly rare disease in China, where only very few cases with sufficient evidences and clinical data have been reported up to now. Here we reported a young patient, onset of hematemesis and melena, who had striking portal hypertension but without liver function damage. Computer tomography scans showed hepatosplenomegaly, intra-hepatic bile ducts dilation, thickening portal vein and tortuous spleen vein, and medullary sponge kidney. Liver biopsy found significant fibrosis in the portal area and ectasia of bile ductules. With sufficient radiologic and pathologic data, our case revealed the features of congenital hepatic fibrosis associated with medullary sponge kidney.

## Introduction

Congenital hepatic fibrosis (CHF) is a developmental disorder that belongs to the family of hepatic ductal plate malformations and is characterized histologically by a variable degree of periportal fibrosis and irregularly shaped proliferating bile ducts [1]. The morbidity of this disease is very low, especially in China. It is an important cause of portal hypertension in the infantile and juvenile age groups. It has been reported that in appoximately half cases of congenital hepatic fibrosis renal cystic disease occurs with varying degree of severity, such as autosomal dominant polycystic kidney disease (ADPKD) and medullary sponge kidney disease [2]. Compared with ADPKD, medullary sponge kidney disease is not predominant among of the associated kidney disease. But in our department, we received a miserable young patient of congenital hepatic fibrosis associated with medullary sponge kidney, who had poor renal function and severe portal hypertension but normal liver function. We excluded the existence of Budd-Chiari syndrome by computer tomographic angiography. Meanwhile, CT scans showed us some interesting images, such as intra-hepatic bile duct dilation, hepatospenomegaly and medullary sponge kidney. Thus we undertook liver biopsy that helped us establish the final diagnosis-congenital hepatic fibrosis (CHF). We reviewed lots of literatures and found exceedingly rare cases had been reported previously in China. In this report, we summarized the clinical manifestations, pictures of CT scan and pathologic observations in order to arouse physician’s attention and provide a little evidence to help clinicians diagnose this disease.

## Case Report

**Figure 1:**
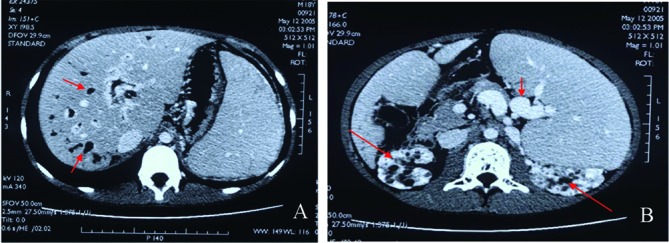
A: Abdominal enhanced CT scan, dilated intra-hepatic bile ducts (red arrow) and hepatosplenomegaly; B: Splenomegaly, circuitous spleen vein (short red arrow), and medullary sponge kidney (long red arrow).

**Figure 2:**
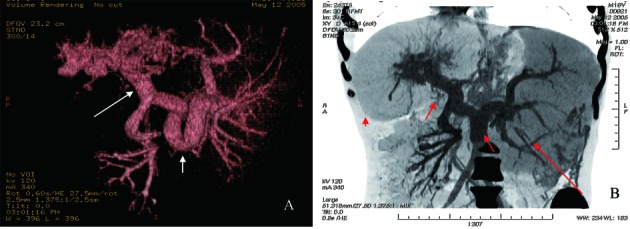
A: CT angiography of visceral vascular, tortuous thickening spleen vein (short white arrow) and proximal portal vein (long white arrow); B: Reconstruction of CT angiography, hepatomegaly (red arrowhead), splenomegaly (long red arrow), circuitous thickening spleen and portal vein (short red arrow).

**Figure 3:**
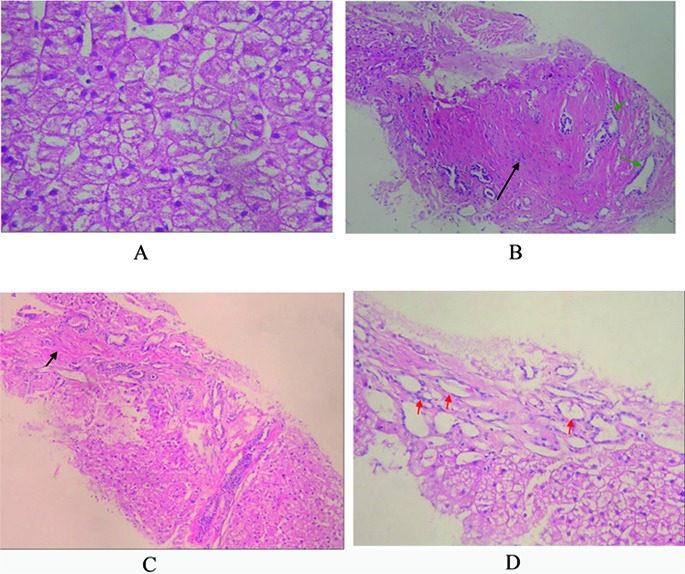
A: Pathology of liver biopsy sample, extensively swelling hepatocytes, and few necrosis hepatocytes. No fat denaturalization and no apoptosis (HE x 200); B: Pathology of liver biopsy sample, significant hyperplasia of fibrous connective tissues (black arrow), and hyperplasia of bile ductules (green arrow). No inflammatory cellular infiltration (HE x 200); C: Pathology of liver biopsy sample: formation of integrated fibrosis interval septum (short arrow) (HE x 100); D: Pathology of liver biopsy sample, dilated hyperplasia of bile ductules (red arrow) and flattening epithelium. No sedimentation of amyloid (HE x 200).

An 18-year-old male patient was admitted to the Department of Hepatogastroenterology, the first affiliated hospital of Dalian Medical University (Dalian city, Liaoning Province, China) because of intermittent hematemesis and melena for 12 days. He had no history of alcohol abuse and hepatitis. And no record of hereditary liver disease was found in his family members. Physical examination showed abdominal wall varices, enlarged liver and spleen. Routine lab biochemistry showed normal levels of aspartate aminotransferase, alanine aminotransferase, albumin, total bilirubin, γ-glutamyltransferase, but elevated concentration of serum creatinine (Cr, 224 µmol/L) and blood urea nitrogen (BUN, 10.04 mmol/L). Laboratory tests failed to show any positive Hepatitis B surface antigen or anti-hepatitis C virus antibody. Blood sample test showed decreased amounts of white blood cells (WBC, 1.54 x 10^9^/L), red blood cells (RBC, 3.0 x 10^12^/L), hemoglobin(Hb, 8.80 g/dL) and platelets (PLT, 32 x 10^9^/L),which indicated the existence of hypersplenism. Immunology test showed normal levels of serum ferrum and ceruloplasmin. Autoimmune antibodies associated with liver disease, such as anti-nuclear antibody, smooth muscle antibody, liver-kidney cytoplasm antibody etc, are all negative. K-F ring was not found with split lamp. Upper barium X-ray exam revealed esophageal varices. Abdominal enhanced CT scans showed hepatosplenomegaly, thickening spleen vein and portal vein, dilated intra-hepatic bile ducts and medullary sponge kidney (Fig. 1A, B). CT angiography revealed dilated circuitous portal and spleen vein, unobstructed hepatic vein and inferior vena cava (Fig. 2A, B). We also undertook liver biopsy, and the noodle-cutting fresh liver tissue was fixed in formaldehyde solution, embedded in paraffin. Sections of 4 µm in thickness were prepared and stained with hematoxylin and eosin. Microscopically, the section sample showed swelling hepatocytes, grossly hyperplasia of fibrous connective tissues in the portal area, bile ductules hyperplasia and intra-hepatic bile duct ectasia, without inflammatory infiltration and sedimentation of amyloid, ferrum and copper (Fig. 3A, B, C, D).

## Discussion

Congenital hepatic fibrosis is a rare autosomal recessive disease named by Kerr in 1961[3]. It is exceedingly uncommon in China mainland. Most patients represent as hepatosplenomegaly, hematemesis or hematochezia. Clinically, liver function is mildly damaged or normal because few hepatocytes are involved. Over half of the patients with CHF have associated renal disease, such as medullary sponge kidney or most commonly autosomal recessive polycystic kidney disease.

Some distinct CT features were frequent in congenital hepatic fibrosis, such as hepatomegaly, varices,splenomegaly, associated ductal plate malformations, and renal abnormalities. The combination of these CT signs is very important for the diagnosis of congenital hepatic fibrosis [4].

Pathologic changes include massive hyperplasia of fibrous connective tissue in portal area, hyperplasia of bile ductules, and/or cholangiectasis [5]. No inflammation or regeneration occurs [6]. The primary change of CHF may be hyperplasia of bile ductules, and then fibrosis in the portal area leads to portal hypertension. Pathologic findings of congenital hepatic fibrosis are considered specific but not sensitive. Typically, patients with CHF do not have cirrhosis and maintain normal hepatic lobular architecture with normal hepatic function.

In this case, we established the diagnosis on the basis of evidences as below: 1) Juvenile onset and variceal bleeding; 2) Normal liver function unparalleled with the severity of portal hypertension; 3) Intra-hepatic bile duct dilation, hepatosplenomegaly and medullary sponge kidney; 4) Histopathologically, hyperplasia of fibrous connective tissues and bile ductules in portal area, and ectasia of bile ducts; 5) Excluded diagnosis of hepatitis, alcohol abuse, metabolic and hereditary liver disease, and autoimmune liver disease. The treatments of CHF include splenectomy, spleen embolism, surgical portosystemic shunt, transjugular intrahepatic portosystemic shut (TIPSS) or liver transplantation [7, 8]. We prescribed omeprazole and somatostatin intravenously for this young patient to control the variceal bleeding. Because of concurrent poor kidney function, we also recommended combined liver and kidney transplantation.

Hitherto the relation between CHF, Caroli’s disease and infant polycystic kidney disease have been undefined yet. The bile ducts involved in Caroli’s disease are bigger and segmental [9]. But in CHF, the smaller and peripheral bile ducts are involved. Clinically and pathologically, an overlap exists between these two diseases: Caroli’s disease and CHF. Some authors summarized Caroli’s disease, CHF and polycystic kidney disease into a clinical syndrome: fibropolycystic liver disease [10].
